# Dr. C. S. Smith on Arthur's Treatment

**Published:** 1874-12

**Authors:** 


### ARTICLE II.

### *Dr. C. S. Smith on Arthur's Treatment.*

I object to the method of treatment advocated by Dr. Arthur, because I consider that it involves serious difficulties, both in theory and practice. It is true that Dr. Arthur tells us that his theory is based on the results of twenty-five years of practice of the method. But twenty or twenty-five years ago the teeth of adults were of a far better quality than those now presented for treatment, particularly in the younger portion of our patients; we are constantly finding the teeth of children seven to nine years of age so much decayed as to be beyond the reach of this practice.

We are too apt, in this as well as in other matters, to observe a fact, and then another one co-existent with or following it, and forthwith to conclude that the one is the cause of the other. If I may make use of a Latin term without being pedantic, he would say that we mistake a *post hoc* for a *propter hoc*, or a thing which happens *after* another, for a thing which happens *on account of* another. "A tooth has been filed; that tooth does not decay, therefore filing is a proper practice." This is superficial and illogical.

I would not change the shape of the tooth materially. It is our duty to restore the form that nature gave; if we destroy the shape of the teeth, we destroy in a measure the usefulness of the organs. It has been said that if it is good practice for the anterior teeth, it is also good for back teeth. This by no means follows; indeed, it is far from the case; for the anterior teeth may be very considerably cut away, without interfering materially with their external appearance, or their effectiveness in incising or articulating. If we follow the practice in the back teeth, we reduce the denture from a set of mill-stones to a row of pegs.

A further objection to this method is, that it requires conditions to its success, that are quite impracticable. The children must be presented at the earliest age, and at stated times, and the appointments, made weeks or months in advance, must be kept, or the system is a failure, according to the author. Now, we know that children are, in ordinary practice, rarely presented until after some of their teeth are so far gone that it is next to impossible to save them. If we who practice filling teeth in place of filing, could see our patients as early and as regularly as Dr. Arthur requires, the loss of teeth with us would be of rare occurrence.

That little skill is required in this practice is claimed by the author, but this can not be true; the file in injudicious hands is a most dangerous instrument, and a good degree of skill must be necessary to carry this method into execution.

Another impracticable thing required is absolute cleanliness; the author admits that unless floss silk, &c., are used daily, or oftener, the system will fail. Now who of *us* is there, knowing as we do that we are dependent on cleanliness to save our teeth, that uses floss silk or rubber dam between his teeth after every meal, or anything approximating to it? We don't do it, and our patients, or a majority of them, won't do it.

